# Minieditorial: Fragilidade em Pacientes não Idosos Submetidos à Cirurgia Cardíaca

**DOI:** 10.36660/abc.20200970

**Published:** 2020-10-13

**Affiliations:** 1 Fundação Universitária de Cardiologia do Rio Grande do Sul Porto AlegreRS Brasil Fundação Universitária de Cardiologia do Rio Grande do Sul, Porto Alegre, RS - Brasil

**Keywords:** Fragilidade, Revascularização Miocárdica/cirurgia, Valvas Cardíacas/cirurgia, Cuidados Pós-Operatórios

A fragilidade é reconhecida como uma síndrome geriátrica caracterizada por um excesso de vulnerabilidade a estressores, com baixa capacidade de manter ou recuperar a homeostase após um evento desestabilizador.^1^ A análise da fragilidade é um assunto bem conhecido e estudado em pacientes idosos, tendo relação direta com o prognóstico e até mesmo com medidas a serem instituídas pré-procedimento para melhorar a qualidade de vida e a evolução dos pacientes.

Apesar da descrição original restringir o termo a pacientes com mais de 65 anos,^2^ a síndrome também atinge pacientes mais jovens.^3^^,^^4^ A fragilidade representa mais aspectos biológicos e fenotípicos do que a idade em si,^3^ e os precursores da síndrome já aparecem precocemente.^3^

O diagnóstico e a terapia da síndrome têm sido quase exclusivamente limitados aos pacientes com mais de 65 anos de idade.^5^^,^^6^ Poucos estudos que analisaram fragilidade incluíram pacientes com menos de 65 anos.^7^^,^^8^ Os fatores classicamente relacionados à fragilidade são idade avançada, baixa escolaridade, tabagismo, uso de terapia de reposição hormonal, ser solteiro/a, ter depressão, baixo nível intelectual e, nos Estados Unidos, ser de etnia Afro-americana ou Espanhola.^9^^,^^10^

A fragilidade está associada ao aumento da mortalidade geral e também prediz piores desfechos em receptores de transplante renal, cirurgia geral e cirurgia cardíaca.^11^^–^^13^ A ferramenta mais utilizada para definir fragilidade são os critérios de fragilidade de Fried, que definem como pré-frágil quem atende a 2 critérios e como frágil quem atende a 3 ou mais dos seguintes critérios:^2^ Perda de peso (≥5 por cento do peso corporal no último ano), exaustão (resposta positiva às perguntas sobre o esforço necessário para realizar atividade), fraqueza (diminuição da força de preensão), velocidade de caminhada lenta (velocidade de marcha) (> 6 a 7 segundos para caminhar 4,5 m) e diminuição da atividade física (Kcal gastas por semana: homens gastando <383 Kcal e mulheres <270 Kcal).

Quando um procedimento cirúrgico precisa ser indicado, instantaneamente uma série de fatores vêm à mente. O momento certo, o risco cirúrgico e o prognóstico do paciente com e sem o procedimento. Os escores de risco analisam a parte orgânica através de dados objetivos, associados ao tipo de cirurgia proposta. No entanto, muitas vezes nos deparamos com valores extremamente baixos, que, subjetivamente, sabemos não serem confiáveis.

Em pacientes idosos com estenose valvar aórtica, os escores de fragilidade já são rotineiramente incorporados à avaliação do risco cardiovascular, auxiliando na indicação ou não da troca transcateter.^14^ Em uma era que enfatiza os custos na Medicina, identificar os pacientes mais vulneráveis, decidir um curso de terapia apropriado e direcionar recursos valiosos são prioridades importantes.^15^

O presente estudo^16^ revela que a análise da fragilidade não está relacionada às comorbidades, fração de ejeção e capacidade funcional, um fato bastante relevante. Outro ponto que deve ser considerado é que por razões metodológicas, a análise não incluiu pacientes com problemas ortopédicos ou neurológicos, com classe funcional IV ou IAM recente, ou em uso de corticoides – nesse contexto, o risco provavelmente seria exponencial. A mortalidade hospitalar foi significativamente maior em pacientes frágeis (29,4%, p = 0,026) do que em pacientes pré-frágeis (8,6%) e não-frágeis (0%).

O estudo engloba uma série de características que o tornam relevante. Primeiramente, trata-se de um tema comum e ainda pouco explorado. Em segundo lugar, permite-nos estimar objetivamente o quanto a fragilidade contribui para o desfecho de pacientes não idosos frágeis submetidos à cirurgia cardíaca, independentemente do tipo. Por fim, chama a atenção dos médicos para a necessidade de incorporar os escores de fragilidade à sua rotina para melhor estratificar e até mesmo definir quando um procedimento deve ou não ser indicado, dando ao médico, paciente e familiares ferramentas que auxiliam na tomada de decisão. TAVI ou cirurgia convencional? CRM ou ACTP? Espero que tenhamos a força de evidência apropriada para indicar aos nossos pacientes o procedimento com a melhor relação risco-benefício.
