# Supplementary material for: Minieditorial: Fragilidade em Pacientes não Idosos Submetidos à Cirurgia Cardíaca
Source: Arq Bras Cardiol. 2020 Oct 13;115(4):611–2. [Article in Portuguese] doi: 10.36660/abc.20200970 (PMC8386969; doi:10.36660/abc.20200970)
Supplement: Translation

# Translation: Short Editorial: Frailty among Non-Elderly Patients Undergoing Cardiac Surgery

**Affiliations:** 1 Porto AlegreRS Brazil Fundação Universitária de Cardiologia do Rio Grande do Sul, Porto Alegre, RS - Brazil

**Keywords:** Frailty, Myocardial Revascularization/surgery, Heart Valves/surgery, Postoperative Care/mortality

Frailty is recognized as a geriatric syndrome characterized by an excess of vulnerability to stressors, with a low ability to maintain or recover homeostasis after a destabilizing event.^1^ The analysis of frailty is a well-known and studied subject in elderly patients, having a direct relationship with the prognosis and even with measures to be instituted pre-procedure aiming to improve the quality of life and the outcome of the patients.

Despite the original description restricting the term to patients older than 65 years,^2^ the syndrome also affects younger patients.^3^^,^^4^ Frailty represents more biological and phenotypic aspects than age itself,^3^ and precursors of the syndrome appear at a young age.^3^

The diagnosis and therapy of the syndrome has been almost exclusively limited to patients over 65 years of age.^5^^,^^6^ Few studies that analyzed frailty included patients under 65 years old.^7^^,^^8^ Factors classically related to frailty are advanced age, low educational level, smoking, use of hormone replacement therapy, not being married, depression, low intellectual level and, in the United States, being of African-American or Spanish ethnicity.^9^^,^^10^

Frailty is associated with an increase in overall mortality and also predicts worse outcomes in kidney transplant recipients, general surgery and cardiac surgery.^11^^–^^13^ The most used tool to define fragility is Fried frailty criteria, which define as pre-fragile those who meets 2 criteria and as fragile those who meets 3 or more of the following criteria:^2^ Weight loss (≥5 percent of body weight in last year), exhaustion (positive response to questions regarding effort required for activity), weakness (decreased grip strength), slow walking speed (gait speed) (>6 to 7 seconds to walk 15 feet) and decreased physical activity (Kcals spent per week: males spending <383 Kcals and females <270 Kcal).

When a surgical procedure has to be indicated, instantly a series of factors comes to mind: the right moment, the surgical risk and the patient's prognosis, with and without the procedure. The risk scores analyze the organic part through objective data, associated with the type of surgery proposed. However, we are often faced with extremely low values, which, subjectively, we know are not reliable.

In elderly patients with aortic valve stenosis, frailty scores are already routinely incorporated into the assessment of cardiovascular risk, helping in the indication or not of transcatheter exchange.^14^ In an era that emphasizes costs in Medicine, identifying the most vulnerable patients, deciding an appropriate course of therapy, and targeting valuable resources are important priorities.^15^

The present study^16^ reveals that the analysis of frailty is not related to comorbidities, ejection fraction and functional capacity, a very relevant fact. Another point that one must consider is that due to methodological reasons, the analysis did not include patients with orthopedic or neurological problems, with functional class IV or recent AMI, or those using corticosteroids - in this context, the risk would probably be exponential. The hospital mortality was significantly higher in frail patients (29.4%, p=0.026) than in pre-frail (8.6%) and non-frail patients (0%).

The study encompasses a series of characteristics that make it relevant. First, it deals with a common and still little explored topic. Second, it allows us to objectively estimate how much frailty contributes to the outcome of fragile, non-elderly patients undergoing cardiac surgery, regardless of the type. Finally, it draws the attention of physicians to the need to incorporate frailty scores into their daily routines in order to better stratify and even define when a procedure should or should not be indicated, providing the doctor, patient and family with tools that assist in the decision-making. TAVI or conventional surgery? CABG or PTCA? I hope that we have the appropriate strength of evidence to indicate to our patients the procedure with the best risk-benefit ratio.
